# Effects of Carbon Tax on Urban Carbon Emission Reduction: Evidence in China Environmental Governance

**DOI:** 10.3390/ijerph20032289

**Published:** 2023-01-27

**Authors:** Aiwen Zhao, Xiaoqian Song, Jiajie Li, Qingchun Yuan, Yingshun Pei, Ruilin Li, Michael Hitch

**Affiliations:** 1 College of Finance, Institute of High-Quality Development in Huaihai Economic Zone, Xuzhou University of Technology, Xuzhou 221018, China; 2 China Institute of Urban Governance, Shanghai Jiao Tong University, Shanghai 200030, China; 3 School of International and Public Affairs, Shanghai Jiao Tong University, Shanghai 200030, China; 4 Key Laboratory of Ministry of Education for Efficient Mining and Safety of Metal Mines, School of Civil and Resource Engineering, University of Science and Technology Beijing, Beijing 100083, China; 5 School of Foreign Languages, Xuzhou University of Technology, Xuzhou 221018, China; 6 School of Political Science and Economics, Yonsei University, Seoul 03722, Republic of Korea; 7 Western Australian School of Mines: Minerals, Energy and Chemical Engineering, Curtin University, Perth, WA 6845, Australia

**Keywords:** carbon peak and carbon neutralization, carbon tax, macroeconomy, carbon emission reduction, effect measurement

## Abstract

Carbon tax is an important economic instrument in achieving the goal of carbon emission reduction and sustainable development. This paper investigates the effects of carbon tax on carbon emission reduction in China. First, a non-competitive input–output table for Carbon Emissions of 28 sectors in China after Carbon Tax was established, based on the “2018 China Non-competitive Input–Output Table (42 Sectors)” and the carbon emission data of sectors provided by China Carbon Emission Accounts and Datasets (CEADs). Then, an input–output price model was established to study the changes on product price, GDP, employment, and carbon dioxide emissions of 28 sectors after carbon taxing ranged from 10 to 200. When the carbon tax rate reaches 200 yuan/ton, the inflation rate will be 5.907%, the total GDP will be decreased to 1.910%, the total labor force will be decreased to 1.744%, and the total carbon emission reduction rate will be increased to 8.171%. Results showed that with the increases in carbon tax, the inflation rate was increased, the rate of carbon emission reduction was increased, and the negative effects on GDP and employment were also increased. Suggestions on policy making, such as combination of carbon taxing and carbon trading, dynamic adjustment mechanism, tax neutrality, and forcing active carbon reduction, were proposed to minimize the adverse effect of levying carbon tax. The results from this paper would provide a reference for the policy making on carbon management.

## 1. Introduction

China’s vision is that her carbon dioxide emissions should peak by 2030 and that she becomes carbon neutral by 2060. To achieve this goal, China is facing serious challenges with respect to carbon emission reduction. As important economic tools, fiscal and taxation policies play important roles in carbon reduction. Many policies have been issued for achieving the goal of carbon peak and carbon neutrality, which mentioned the importance of carbon tax [1]. At present, some tax or preferential tax policies related to carbon emission reduction are being implemented in China, but their promotion effect on achieving the goals of carbon peak and carbon neutrality proves limited, for they are not originally designed to regulate carbon emissions directly, and therefore the carbon tax should become a new source of taxation for energy conservation and carbon emission reduction [2]. According to the World Bank, by 2021 carbon taxes have been implemented in more than 30 countries and regions around the world, mainly in Europe and some developed countries [3]. The carbon trading market was set up in 2021, but the carbon tax was not imposed.

At present, several studies related to carbon taxing have been investigated, with some researchers invested in the correlation between carbon taxes and carbon trading. In terms of comparative research on carbon taxes and carbon trading policies, the carbon tax was considered to have originated from the Pigouvian tax [4], while emissions trading was considered to have originated from the Coase theorem [5]. The reform of the European Union’s carbon border regulation mechanism (CBAM), based on Gerberi theory, has also made significant progress [6]. As for the function mechanism of carbon tax policy, the carbon tax system focuses on price control [7,8] while carbon trading is based on total emission control [9,10,11,12]. From the perspective of the emission reduction effect, carbon trading with the high certainty of carbon emission reduction belongs to the means of total amount control [13,14], while carbon tax is a price means, which plays an indirect role, enabling enterprises to have stable expectations for increased costs due to fixed tax rates. As far as the aspect of collaborative research on carbon tax and carbon trading policies, most researchers believe that with China’s current situation, all the problems of carbon emission reduction in China cannot be solved by only relying on carbon trading. They suggested that the Chinese government should begin to levy a carbon tax at the right time [15,16,17,18], and formulate a reasonable carbon tax system [19,20], thus forming a compound carbon emission model that combines carbon tax and carbon emission trading systems [21,22,23]. They also proposed that China should choose the incorporated legislative model of a carbon tax, dynamically adjust the rate level of carbon tax according to the change of social carbon cost [24,25,26,27], construct the coordination mechanism of carbon tax and carbon trading [28,29,30], and clearly divide the respective regulatory scope of carbon tax and carbon trading.

Additionally, the effects of carbon tax policy from different perspectives were investigated by several researchers. In terms of the emission-reduction effect of carbon tax, the effectiveness of carbon tax in reducing carbon emissions is often demonstrated [31,32,33,34,35], but some studies, instead, argue that carbon tax has not actually achieved the effect of its emission reduction [36]. In research on the economic effect of carbon tax, most scholars think that the negative macroeconomic effect of carbon tax does exist [37,38,39,40], leading to diminishing distribution effects [41,42,43,44] which can be controlled within a reasonable range through a reasonable design of tax rate [45,46]. Some scholars hold the opinion that the negative impact of carbon tax on economic growth is not so great, and the appropriate complementary systems will have a positive impact on the economy [47,48]. Others argue that carbon tax refunds can reduce the negative macroeconomic impact [49]. As for the research on the double dividend effect of carbon tax, Pearce (1991) formally put forward the concept of the “double dividend” of carbon tax for the first time [50]. Goulder (1995) distinguished between a “weak double dividend” and a “strong double dividend” [51]. Most of the research results found by foreign scholars support the establishment of “double dividends” under certain conditions [52,53,54]. With research, some scholars also believe that carbon tax has a significant negative impact on economic growth, household consumption, and corporate income, and the “double dividend” effect of carbon reduction and promotion of growth cannot be achieved in a short term [55,56,57,58,59]. Therefore, it is crucial to clarify the connection between carbon tax and macroeconomic developments.

To sum up, progress has been made in existing research, but there is still some work to be completed to measure the effects of different carbon tax rates on economic growth, employment, and carbon emission reduction in different sectors. In view of this, this study compiles “2018 Non-competitive Input-Output Tables of China for Carbon Emissions after Carbon Tax (28 Departments)” based on the research of other scholars. Additionally, with the help of input–output price change models, the effects of different carbon tax rates on prices, GDP, employment, and carbon emission reduction in 28 sectors are explored from the perspectives of “added value” and “final use”. The results of this study will provide a theoretical basis and practical path for the levying of carbon taxes.

## 2. Materials and Methods

The carbon tax is ultimately a price issue. In the process of production, products need to be mutually consumed and supplied. The changes in the price of products in one sector could affect the price of products in the other sectors. Thus, the relationship between the products of various sectors are complicated. This relationship of mutual influence could be calculated using the input–output mode [60], which reflects the internal relationship among various sectors of the national economy and various production links. The input–output mode could simulate the results of various economic policies when using prices, labor remuneration, tax revenue, etc., as exogenous control variables [60].

The input–output model could calculate the impact of changes in added value (e.g., GDP, tax revenue) on prices, and comprehensively analyze the relationship among added value, production, and consumption from the perspective of the national economy. This study only considers the impact of different carbon tax rates on the sector prices, sector GDP, sector employment, and sectoral carbon emission reductions. Many software packages are available for input–output analysis. In this study, MATLAB 2020 and EXCEL 2019 were selected due to their relative ease of use.

### 2.1. Non-Competitive Carbon Emission Input-Output Table

The intermediate use and final use in the Competitive Input–Output Table include both national products and imports. Since this study only investigated the carbon emissions of domestic products, it is necessary to use the “Non-competitive Input–Output Table” that distinguishes between domestic products and imports.

Combining the “2018 China Non-competitive Input–Output Table” and the “China Energy Statistical Yearbook 2019”, this paper compiled “2018 Non-competitive Input–Output Tables of China for Carbon Emissions after Carbon Tax (28 Departments)” (Table 1). Four energy sectors are identified as follows: coal mining and dressing industry, petroleum and natural gas extraction industry, refined petroleum and nuclear fuel processing industry, as well as gas production and supply industry. In energy statistics, the production and supply of electric power and heat are often regarded as secondary energy sectors. The carbon emissions implied in the use of secondary energy, such as electricity and heat, are accounted for as indirect carbon emissions. However, these secondary energy sectors are not listed as energy sectors in the “carbon emissions” input–output table in the paper, since the direct carbon emissions of the products are usually counted as zero by the terminal departments.

As can be seen from Table 1, the net production tax after the carbon tax (T’) is calculated using Equation (1).

(1) $$T^{\prime }=T+T*$$

where T is the net production tax and T* is the carbon tax.

The gross domestic product (GDP) (i.e., the added value in the input–output table) is calculated according to the income method (Equation (2)).

GDP = V + T + D + S(2)

where V is the remuneration of workers, D is the depreciation of fixed assets, and S is the operating surplus.

Therefore, the total added value after the carbon tax (GDP’) is calculated using Equation (3).

(3) $${\mathrm{GDP}}^{\prime }=V+T+T*+D+S$$

Then, the domestic final use after the carbon tax equals the original domestic final use plus carbon tax. Similarly, the total output after the carbon tax equals the original total output pluses carbon tax.

### 2.2. Analysis of the Impact of Carbon Tax

The carbon tax rate is closely related to the basis for taxation. It is based on the quantity and fixed tax rate, because CO_2_ emissions are used as the tax basis, and the ecological damage caused by CO_2_ emissions is directly related to its quantity, not to its value [62].

#### 2.2.1. Impact of Carbon Tax on Price

In the value input–output table, the price index expression of department *j* can be calculated by a column model [60,63].

(4) $$\sum_{i=1}^{n}a_{ij}p_{i}+a_{vj}+a_{tj}+a_{dj}+a_{sj}=p_{j}\;\;\;\;(j=1,2,\ldots ,n)$$

where *p_j_* is the price index of sector *j*.

The matrix form of Equation (4) is as follows:

(5) $$A^{T}P+A_{v}^{T}+A_{t}^{T}+A_{d}^{T}+A_{s}^{T}=P$$

where *P* = (P_1_, P_2_,… P_n_)^T^ is the column vector of product price before carbon tax is levied; *A* is the matrix of direct consumption coefficient; *A^T^* is the transpose matrix; *A_v_* = *v_j_*/*X_jj_* is the row vector for the coefficient of labor remuneration before the carbon tax is levied; *A_d_* = *d_j_*/*X_j_* is the row vector for the depreciation coefficient of fixed assets; *A_t_* = *t_j_*/*X_j_* is the row vector for the net production tax coefficient; and *A_s_* = *s_j_*/*X_j_* is the row vector for the operating surplus coefficient.

From Equation (5) we can obtain the following:

(6) $$P={\left[{\left(I-A\right)}^{-1}\right]}^{T}\cdot (A_{v}^{T}+A_{t}^{T}+A_{d}^{T}+A_{s}^{T})$$

where (*I* − *A*)^−1^ is the Leontief inverse matrix.

Usually, Equation (6) is treated as a product theoretical price model or a general price model.

After the carbon tax is levied, the coefficient of net production tax of sector *j* changes from $a_{tj}$ to $a_{tj}^{*}$, and the price index ($p_{j}^{*}$) becomes the following:

(7) $$\sum_{i=1}^{n}a_{ij}^{}p_{i}^{*}+a_{vj}+a_{tj}^{*}+a_{dj}+a_{sj}=p_{j}^{*}\;\;\;\;(j=1,2,\ldots ,n)$$

The matrix form of Equation (7) is as follows:

(8) $$P^{*}={\left[{\left(I-A\right)}^{-1}\right]}^{T}\cdot (A_{v}^{T}+A_{t}^{*T}+A_{d}^{T}+A_{s}^{T})$$

where P* = ($P_{1}^{*}$, $P_{2}^{*}$,…$P_{n}^{*}$)^T^ is the column vector of product price after carbon tax is levied.

As can be seen by Equations (4) and (7), after the carbon tax is levied, the prices of domestic products in various sectors will also change accordingly since the coefficient of net production tax changes from $a_{tj}$ to $a_{tj}^{*}$. The column vector for the change quantity of product price Δ*P* is as follows:

(9) $$\Delta P_{t}={\left[{\left(I-A\right)}^{-1}\right]}^{T}\cdot A_{t}^{*T}-A_{t}^{T})={\left[{\left(I-A\right)}^{-1}\right]}^{T}\cdot \Delta A_{t}^{T}$$

where, Δ*P_t_* = (ΔP_t1_, ΔP_t2_,…, ΔP_tn_)^T^ is the column vector of product price changes in various sectors before and after the carbon tax is levied; *A_t_* = *t_j_*/*X_j_* is the row vector for the coefficient of net production tax before the carbon tax is levied, $A_{t}^{*}$ = $t_{j}^{*}$/$X_{j}^{*}$ is the row vector for the coefficient of net production tax after the carbon tax is levied; and Δ*A_t_* = (Δa_t1_, Δa_t2_, …, Δa_tn_)^T^ is the row vector for the coefficient of net production tax before and after carbon tax is levied.

Equation (9) is a model for product price varying. For the non-competitive input-output table, the Equation (9) becomes Equation (10).

(10) $$\Delta P_{t}={\left[{\left(I-A^{d}\right)}^{-1}\right]}^{T}\cdot (A^{d*T}-A_{t}^{dT})={\left[{\left(I-A^{d}\right)}^{-1}\right]}^{T}\cdot \Delta A_{t}^{dT}$$

where *A^d^* is the matrix for the direct consumption coefficient of domestic products.

The consumer price index after carbon tax ($CPI^{*}$) is calculated by Equation (11).

(11) $$CPI^{*}=\frac{{\left(P^{*}\right)}^{T}\cdot Y_{CP}^{*}}{P^{T}\cdot Y_{CP}}$$

where, *Y_CP_* is the column vector for final consumption demand of residents before the carbon tax is levied; $Y_{CP}^{*}$ is the column vector for final consumption demand of residents after the carbon tax is levied; *P* is the column vector of price before the carbon tax is levied; and P* is the column vector of price after the carbon tax is levied.

The growth rate of the consumer price index after the carbon tax is the inflation rate IR [64], which is expressed as Equation (12).

(12) $$IR=\frac{CPI^{*}-CPI}{CPI}=CPI^{*}-1=\frac{{\left(P^{*}\right)}^{T}\cdot Y_{CP}^{*}}{P^{T}\cdot Y_{CP}}-1$$

where *CPI* is consumer price index before carbon tax.

The inflation rate = $CPI^{*}$ − 1.

#### 2.2.2. The Impact of a Carbon Tax on GDP

As can be seen from Table 1, the gross domestic product (i.e., added value) before the carbon tax (*GDP*) is as follows:

(13) GDP=V+T+D+S

Gross domestic product after a carbon tax (GDP*) is as follows:

(14) $${\left(GDP\right)}^{*}=V+T+T^{*}+D+S$$

Taking into account the inflation, the gross domestic product after the carbon tax is as follows:

(15) $$GD{P*}^{\prime }=\frac{GDP^{*}\cdot I}{I^{T}\cdot CPI^{*}}$$

where GDP*′ is the gross domestic product with inflation after the carbon tax is levied and *I* = (1, 1,…1)^T^ is the column vector.

Taking into account the inflation, the rate of change for *GDP* after the carbon tax ((Δ*GDP*)*′) derived from Equations (13)–(15) is as follows:

(16) $${(\Delta GDP)*}^{\prime }=\frac{GD{P*}^{\prime }\cdot I-GDP\cdot I}{GDP\cdot I}$$

Due to the levy of carbon tax, the *GDP* of each sector decreases as the rate of carbon tax increases, resulting in a decrease in the total *GDP*.

#### 2.2.3. The Impact of Carbon Tax on Employment

Since price changes in all sectors are inevitably incurred by the levy of carbon tax, this will, in turn, lead to changes in the output in various sectors and the changes of *GDP* of each sector. As can be seen from Table 1, workers’ remuneration occupies a great portion of *GDP*, assuming that the proportion of workers’ remuneration to *GDP* remains unchanged before and after the carbon tax is levied, as is shown in Equation (17):

(17) $$\frac{V\cdot I}{GDP\cdot I}=\frac{V^{*}\cdot I}{GDP^{*}\cdot I}=\frac{{V*}^{\prime }\cdot I}{GD{P*}^{\prime }\cdot I}$$

where *V* is the remuneration of workers after the carbon tax; V* is the remuneration of workers before the carbon tax; and V*′ is the remuneration of workers after the carbon tax on the premise of inflation.

In view of inflation, the actual labor remuneration will inevitably decrease after the carbon tax is levied on the premise that the proportion of workers’ remuneration in *GDP* remains unchanged, which will lead to a change in the number of laborers; that is, the levy of carbon tax will have an impact on employment because of the following:

Number of workers = Remuneration of workers/Average worker’s remuneration(18)

Considering the inflation, the rate of change in the workforce after the carbon tax ((Δ*L*)*′) is the following:

(19) $$\Delta {L*}^{\prime }=\frac{{L*}^{\prime }\cdot I-L\cdot I}{L\cdot I}$$

where *L* is the number of laborers before the carbon tax, L* is the number of laborers after the carbon tax, and L*′ is the number of laborers after the carbon tax on the premise of inflation.

#### 2.2.4. The Impact of Carbon Tax on Carbon Emissions Reduction

Assuming that the intensity of carbon emission remains unchanged before and after the carbon tax is levied, that is, the carbon emission per unit of *GDP* will remain unchanged before and after carbon tax is levied, the following is the case:

(20) $$\frac{CO_{2}\cdot I}{GDP\cdot I}=\frac{{\left(CO_{2}\right)}^{*}\cdot I}{{\left(GDP\right)}^{*}\cdot I}=\frac{{(CO_{2})*}^{\prime }\cdot I}{{(GDP)*}^{\prime }\cdot I}$$

where *CO*_2_ is the carbon dioxide emissions before carbon tax; (*CO*_2_)* is the carbon dioxide emissions after carbon tax; and (*CO*_2_)*′ is carbon dioxide emissions after carbon tax on the premise of inflation.

Taking the inflation into account, the rate of change in carbon dioxide emission after the carbon tax ((Δ*CO*_2_)*′) is the following:

(21) $${(\Delta CO_{2})*}^{\prime }=\frac{{(CO_{2})*}^{\prime }\cdot I-CO_{2}\cdot I}{CO_{2}\cdot I}$$

### 2.3. Data Source

Given the availability of data, the carbon dioxide emissions in this paper include only carbon dioxide emissions from fossil energy consumption in various sectors, excluding other greenhouse gas emissions. The data on input-output in the paper are from the “2018 Non-competitive Input–Output Tables of China”, and the data on carbon emission are from the China Carbon Emission Accounts and Datasets (CEADs) [65].

## 3. Results

With the equations mentioned in Section 2, simulation is made by EXCEL software and MATLAB software when carbon tax rate is T* = 10 CNY/ton~T* = 200 CNY/ton. The results of price-effect measurement, economic effect measurement, employment-effect measures and reduction-effect measurement of carbon taxes were obtained.

### 3.1. Price-Effect Measurement of Carbon Tax

**Figure 1:**
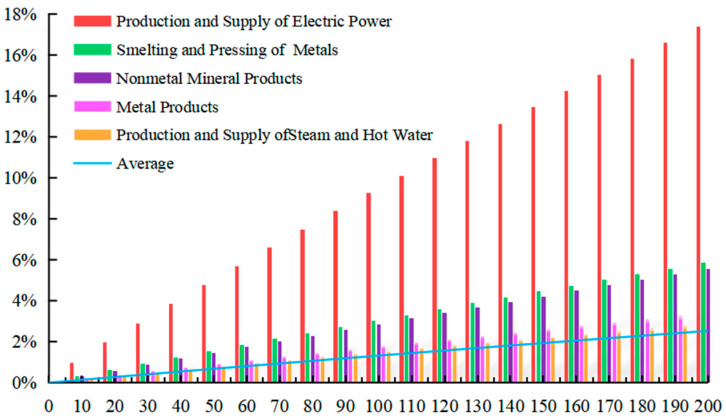
Relations of carbon tax and sectoral domestic products price.

With Equation (10), the simulation is made by EXCEL software and MATLAB software, when the carbon tax rate is T* = 10 CNY/ton~T* = 200 CNY/ton, displaying that carbon tax rate has a different impact on the price changes of domestic products from 28 sectors, among which the top 5 sectors with the greatest impact and average impact on 28 sectors are shown in Figure 1.

When the carbon tax is levied, the product prices of 28 sectors will all rise to varying degrees. Taxes on carbon dioxide result in higher prices and higher costs in energy-intensive sectors. The price rise will be transmitted to other sectors through input–output relationships between production sectors. Thus, it results in cost-driven inflation. With the increase in carbon tax rate from 10 CNY/ton to 200 CNY/ton, the prices of various sectors will also rise. When the carbon tax rate is T* = 200 CNY/ton, the average price of 28 sectors will rise by 2.524%. Among them, the sector of Production and Supply of Electric Power is greatly affected, with the price rising by 17.382%. The sector of Smelting and Pressing of Metals ranked in second place, with the price rising by 5.844%. The sector of Nonmetal Mineral Products ranked third, with the price rising by 5.546%. A total of 7 of the top 10 sectors whose price changes of products are greatly affected after carbon tax is levied are heavy industries in the secondary industry. It is mainly due to the fact that China’s current economy is in the stage of heavy chemical industry. Therefore, the levy of carbon tax has little impact on the prices of the primary industry and the tertiary industry.

The carbon tax leads to the rise in product prices in various sectors, because no matter whether it is levied on producers or consumers, the tax burden is ultimately shared by producers and consumers due to the tax burden shifting. After the carbon tax is levied, the price paid by consumers will be raised, and the production cost of producers will also be raised. Consequently, the increase in production costs will lead to cost-driven inflation, which results in the price rising of all the sectors to varying degrees.

The impact of different carbon tax rates on inflation can be calculated by Equations (11) and (12), as shown in Table 2.

As can be seen from Table 2, the carbon tax rate continues to increase, and so does the inflation rate. When the carbon tax rate is T* = 10 CNY/ton, the inflation rate will be 0.293%; when the carbon tax rate is T* = 100 CNY/ton, the inflation rate will be 2.942%; and when the carbon tax rate is T* = 200 CNY/ton, the inflation rate will be 5.907%.

As for the sector of high carbon, manufacturers tend to pass the carbon tax on to their product costs through tax burden shifting due to the large carbon emissions and high carbon tax burden, so the levy of carbon tax usually results in cost-driven inflation with the rising prices of products. 

**Figure 2:**
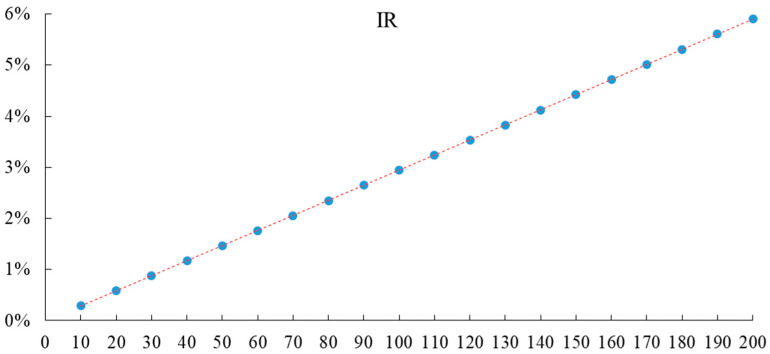
Different carbon tax rates and inflation rates.

Assuming that the carbon tax rate (T*) is the independent variable and the inflation rate (IR) is the dependent variable, an EXCEL scatter diagram of the two is drawn to conveniently facilitate the explanation and the linear relationship between them as shown in Figure 2, where the horizontal axis represents different carbon tax rates, and the vertical axis represents the inflation rate.

The relationship between the carbon tax rate (T*) and the inflation rate of the dependent variable (IR) can be fitted by EXCEL scatter diagram, trend line analysis, and Eviews software (See Equation (22)).

(22) $$\mathrm{IR}=0.000295T*-0.0000907\;R^{2}=0.999996$$

Equation (22) shows that with the increase in carbon tax rate, the inflation rate also increases, and there is a positive correlation between them. Because the intercept term is very small, it can be roughly considered that there is a linear relationship between them. R^2^ is goodness-of-fit with a maximum value of 1, and the closer the value of R^2^ is to 1, the better the regression line fits the observed value [66].

### 3.2. Economic Effect Measurement of Carbon Tax

**Figure 3:**
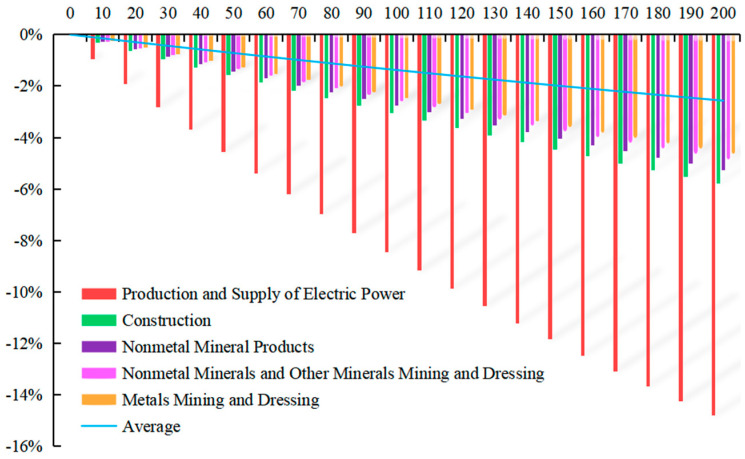
Relationship between carbon tax and sectoral GDP.

With Equation (16), the simulation of carbon tax rate T* = 10 CNY/ton~T* = 200 CNY/ton is made by EXCEL software and MATLAB software, displaying that carbon tax rate has a different impact on the *GDP* changes of 28 sectors, among which the 5 sectors with the greatest impact are shown in Figure 3. The horizontal axis represents different carbon tax rates, and the vertical axis represents the change rate of industrial *GDP*.

When the carbon tax is levied, the *GDP* of 28 sectors will decline to varying degrees. For producers, due to the tax burden shifting, energy becomes a more expensive factor of production, which increases the production cost, and the production possibility curve moves inward, reducing production. For consumers, due to the tax burden shifting, consumers will have to pay higher prices, resulting in lower demand. Consequently, the reduction in production and consumption inevitably leads to a decrease in *GDP*.

With the increase in the carbon tax rate from T* = 10 CNY/ton to T* = 200 CNY/ton, the change rate of *GDP* in various sectors also declines. When the carbon tax rate is T* = 200 CNY/ton, the average change rate of *GDP* in 28 sectors will be −2.567%, among which the top three sectors with the greatest impact are, successively, the sector of Production and Supply of Electric Power and Heating with the change rate of *GDP* by −14.808%, the sector of Construction with the change rate of *GDP* by −5.789%, and the sector of Nonmetal Mineral Products with the change rate of *GDP* by −5.254%.

The impact of different carbon tax rates on changes in total *GDP* can be calculated by Equation (16), as shown in Table 3.

**Figure 4:**
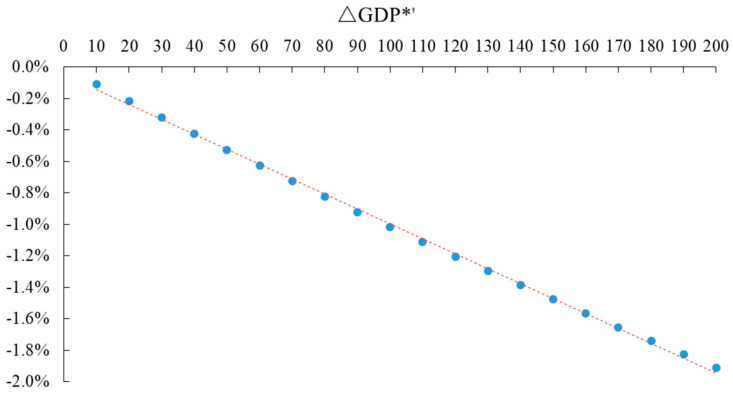
Different rates of carbon tax and the change rate of total GDP.

Assuming that the carbon tax rate (T*) is the independent variable and the change rate of total *GDP*(ΔGDP*′) is the dependent variable, in order to explain the problem, the EXCEL scatter diagram of the two is drawn to reveal the linear relationship between them, as shown in Figure 4. The horizontal axis represents different carbon tax rates, and the vertical axis represents the change rate of *GDP*.

The relationship between the carbon tax rate (T*) and the change rate of the dependent variable *GDP* (ΔGDP*′) can be fitted by an EXCEL scatter diagram, trend line analysis, and Eviews software, as shown in Equation (23).

(23) $${\Delta \mathrm{GDP}*}^{\prime }=-0.0000947T*-0.0005\;R^{2}=0.9989$$

Equation (23) shows that as the carbon tax rate increases, the rate of change in total *GDP* decreases, and the two are negatively correlated. Because the intercept term is very small, it can be roughly assumed that the two are in a linear relationship. R^2^ is the goodness-of-fit, and its maximum value is 1. The closer the value of R^2^ is to 1, the better the fitting of the regression line to what the observed value is.

### 3.3. Employment-Effect Measures of Carbon Taxes

**Figure 5:**
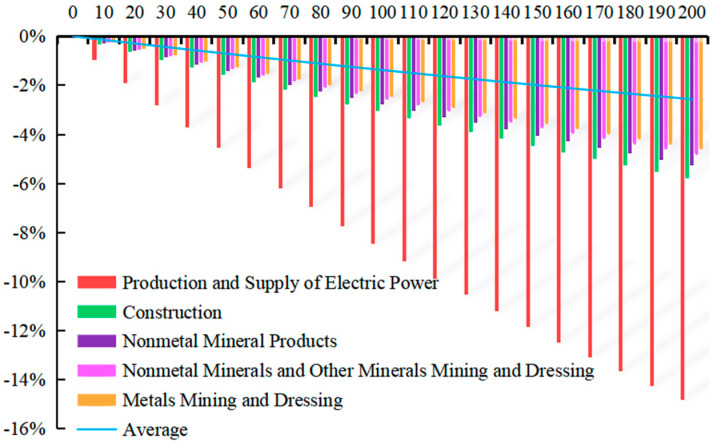
Impact of different carbon tax rates on the change of industrial employment.

With Equations (18) and (19), the simulation under carbon tax rate T* = 10 CNY/ton ~ T* = 200 CNY/ton is made by EXCEL software and MATLAB software, displaying that carbon tax rate has a different impact on the change of employment in 28 sectors, among which the 10 sectors with the greatest impact are shown in Figure 5. The horizontal axis represents different carbon tax rates, and the vertical axis represents the change rate of industrial employment.

When the carbon tax is levied, the number of workers in all 28 sectors will decline to varying degrees. The reason is that after the carbon tax is levied, as an important factor of production, energy tends to become more expensive for the producers of high-carbon enterprises due to the tax burden shift, and the production possibility curve will move inward with the increase in production cost, making production capacity decline to a low level, and the number of laborers inevitably decrease.

With the increase in the carbon tax rate from T* = 10 CNY/ton to T* = 200 CNY/ton, the number of laborers in each sector declines accordingly. When the carbon tax rate is T* = 200 CNY/ton, the average change rate of employment in 28 sectors will be −2.567%, among which the top three sectors are, successively, the sector of Production and Supply of Electric Power and Heating with the change rate of GDP by −14.808%, the sector of Construction with the change rate of GDP by −5.789%, and the sector of Nonmetal Mineral Products with the change rate of GDP by −5.254%.

With Equations (17)–(19), calculations are made on the impact of different carbon tax rates on the changes of the total number of laborers. See Table 4.

**Figure 6:**
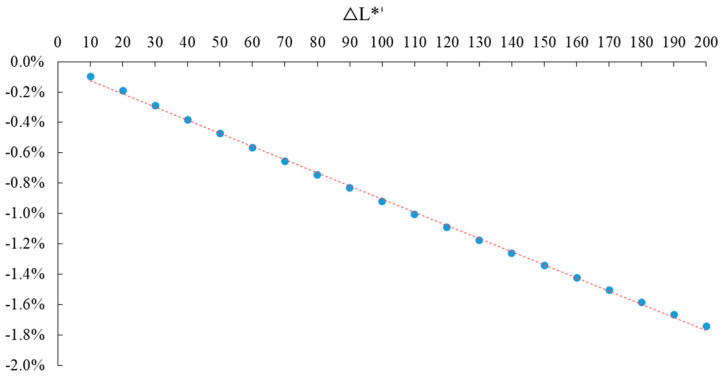
Different rates of carbon tax and the change rate of laborers’ number.

Assuming that the carbon tax rate (T*) is the independent variable and the change rate of laborers’ number is the dependent variable, in order to explain the problem, the EXCEL scatter diagram of the two is drawn to reveal the linear relationship between them, as shown in Figure 6. The horizontal axis represents different carbon tax rates, and the vertical axis represents the change rate of total employment.

The relationship between the rate of carbon tax (T*) and the change rate of employment number can be fitted by EXCEL scatter diagram, trend line analysis, and Eviews software, as shown in Equation (24).

(24) $${\Delta L*}^{\prime }=-0.0000866T*-0.000388\;R^{2}=0.9992$$

Equation (21) shows that as the rate of carbon tax increases, the change rate of laborers’ number decreases, and the two are negatively correlated. Because the intercept term is very small, it can be roughly assumed that the two are in a linear relationship. R^2^ is the goodness-of-fit, and its maximum value is 1. The closer the value of R^2^ is to 1, the better the fitting of the regression line to the observed value.

### 3.4. Reduction Effect Measurement of Carbon Taxes

**Figure 7:**
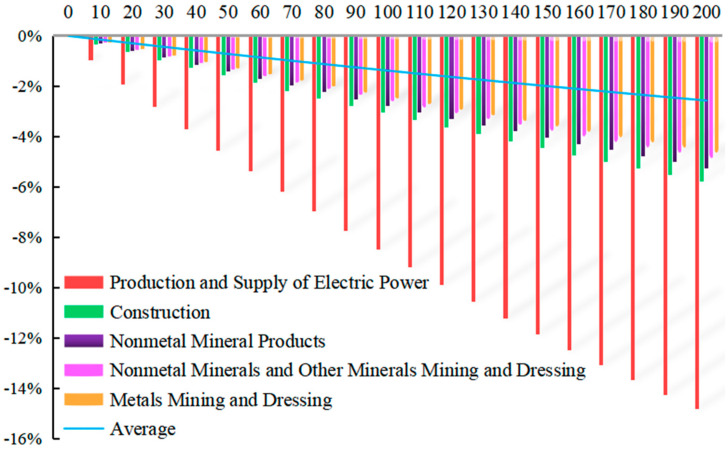
Impact of different rates of carbon tax on the changes of the industrial carbon dioxide emission.

With Equation (15), the simulation under carbon tax rate T* = 10 CNY/ton ~ T* = 200 CNY/ton is made by EXCEL software and MATLAB software, displaying that the rate of carbon tax has a different impact on the change of carbon dioxide emission in 28 sectors, among which the top 10 sectors with the greatest impact are shown in Figure 7. The horizontal axis represents different carbon tax rates, and the vertical axis represents the change rate of industry carbon emissions.

When the carbon tax is levied, the carbon emission volume of 28 sectors will all decline to varying degrees. From the perspective of income effect, for producers, the levy of carbon tax adds to the cost of raw materials, the production possibility curve moves inward, and carbon emission will be inevitably reduced due to reduced production. For consumers, they will have to pay higher prices due to the levy of carbon tax. On the premise that the budgeted revenues remain unchanged, consumers will buy fewer goods, thus leading to the reduction in carbon emissions. From the perspective of the substitution effect, for producers, they will carry out technological innovation to improve energy efficiency, reduce the use of high-carbon energy as a factor of production, find alternative energy sources, and improve other production factors, such as capital and labor, to replace energy. For consumers, they will inevitably reduce their consumption of high-carbon products as producers reduce their production of high-carbon products. The combination of the income effect and the substitution effect will inevitably lead to the reduction in carbon emissions in various sectors.

With the increase in the carbon tax rate from T* = 10 CNY/ton to T* = 200 CNY/ton, the carbon emission of each sector also declines. When the carbon tax rate is T* = 200 CNY/ton, the average volume of carbon emission in 28 sectors will be −2.567%, among which the top three sectors with the greatest impact are, successively, the sector of Production and Supply of Electric Power and Heating with the change rate of carbon emission by −14.808%, the sector of Construction with the change rate of carbon emission by −5.789%, and the sector of Nonmetal Mineral Products with the change rate of carbon emission by −5.254%.

The impact of different carbon tax rates on inflation can be calculated by Equation (21), as shown in Table 5.

**Figure 8:**
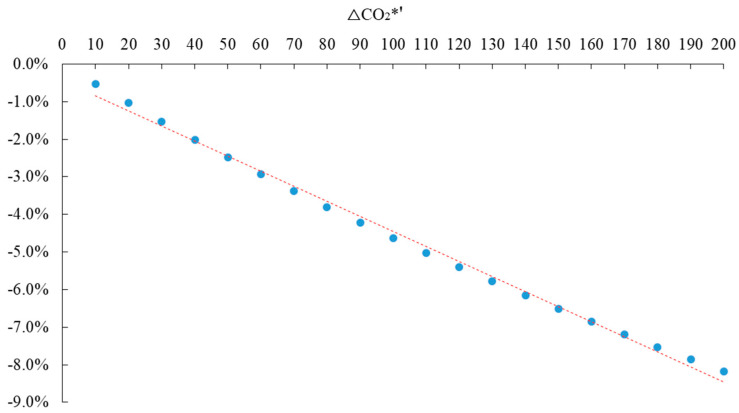
Different rates of carbon tax and the change rate of carbon dioxide emission.

Assuming that the rate of carbon tax (T*) is the independent variable and the change rate of carbon dioxide emission is the dependent variable, in order to explain the problem, the EXCEL scatter diagram of the two is drawn to reveal the linear relationship between them, as shown in Figure 8. The horizontal axis represents different carbon tax rates, and the vertical axis represents the change rate of total carbon emissions.

The relationship between the rate of carbon tax (T*) and the change rate of carbon dioxide emission can be fitted by EXCEL scatter diagram, trend line analysis, and Eviews software, as shown in Equation (25).

(25) $${\Delta {\mathrm{CO}}_{2}*}^{\prime }=-0.0004T*-0.004481\;R^{2}=0.9954$$

Equation (25) shows that as the rate of carbon tax increases, the change rate of carbon dioxide emission decreases, and that the two are negatively correlated. Because the intercept term is very small, it can be roughly assumed that the two are in a linear relationship. R^2^ is the goodness-of-fit, and its maximum value is 1. The closer the value of R^2^ is to 1, the better the fitting of the regression line to the observed value.

## 4. Discussion

The levy of carbon tax is an effective way to reduce carbon dioxide emissions and eliminate climate change [67]. With the compilation of 2018 China’s non-competitive input–output table for Carbon Emissions after Carbon Tax (28 sectors) and the model of input–output product price change, this study mainly investigates the economic effect and carbon emission reduction effect with different carbon tax rates. The results show that without considering technological innovation, the higher the carbon tax rate is, the higher the product price and inflation rate of each sector will be, the faster the *GDP* and the number of laborers will decline, and the more remarkable the emission reduction effect will manifest. In the long run, the carbon tax will force enterprises to carry out technological innovation, which will have a long-term positive effect on economic growth.

To ensure a better function of carbon tax as an economic tool for carbon emission reduction and sustainable development, the following suggestions on policy are proposed.

### 4.1. Combination of Carbon Tax and Carbon Trading

As the results have shown in this paper, when the rate of carbon tax is 200 CNY/ton, the rate of inflation is 5.907%. Accordingly, only using the single economic tool of carbon tax to reduce carbon emissions tends to bring about greater inflation, which is not conducive to social stability [68]. Thus, the combination of carbon tax with carbon trade would be complementary and cooperative with each other, and undoubtedly more effective [69,70]. At present, the combination of carbon tax and carbon trading have been in practice internationally. For example, Denmark and Finland levied carbon taxes, and meanwhile, they joined the EU carbon trading system.

The carbon trading market was fully launched in China in 2021; however, it only covers 50% of China’s carbon emissions. It is difficult to achieve the goal of carbon peak and carbon neutrality by relying on carbon trading alone. Carbon tax is suggested to be levied by the government to make up the gap of carbon trading in China. The combination of carbon tax and carbon emission trading, on the one hand, can achieve the complementarity of coverage and form a more comprehensive carbon-emission-reduction mechanism. On the other hand, it can realize the coordination of the price mechanism and give play to the carbon price stabilization function of the carbon tax.

### 4.2. Establishment of Dynamic Adjustment Mechanism for Carbon Tax Rate

As shown in the Section 3, the higher the rate of carbon tax is, the greater the negative effect on GDP will be. When the rate of carbon tax reaches 200 CNY/ton, the change rate of GDP is −1.910%. The design of a carbon tax rate should reflect the marginal cost of CO_2_ emission reduction to the maximum extent, considering the impact on macro-economy and industrial competitiveness. China should establish a dynamic adjustment mechanism for the carbon tax rate according to the needs of energy conservation and emission reduction at different stages, so as to ensure the effectiveness of carbon tax policy while avoiding increasing the cost of tax collection and management [71]. Based on the results of this paper, differential tax rates should be set considering the impact of carbon tax on inflation and *GDP*. If the inflation rate is controlled within 1.5%, 3%, 4.5%, and 6%, respectively, and the impact on *GDP* is controlled within 0.5%, 1%, 1.5%, and 2%, respectively, the corresponding optimal tax rates are 45 CNY/ton, 95 CNY/ton, 150 CNY/ton, and 200 CNY/ton, respectively.

### 4.3. Keep Tax Neutrality Using Carbon Taxes to Make Less Impact on Employment

As shown in the Section 3, the higher the rate of carbon tax is, the greater the negative effect on employment will be. When the rate of carbon tax reaches 200 CNY/ton, the change rate in the number of laborers is −1.744%. Although the levy of carbon tax usually reduces employment, the reduction in the tax burden of other taxes will, instead, increase employment opportunities [72]. In this field, the successful experience of European and American countries from which China may learn. The United States imposes carbon tax on products with carbon content exceeding the baseline. While Germany’s initial carbon tax rate is relatively low, and the tax rate is gradually increased in stages. Both countries reduce or return other taxes, such as corporate income tax, while imposing carbon tax, so as to avoid the decline in enterprise competitiveness caused by the increase in tax burdens, reduce the impact of carbon taxes on employment, and thus implement carbon tax policies step by step. 

### 4.4. Force Enterprises Shift from Passive to Active Carbon Emission Reduction

As shown in Section 3, the higher the carbon tax rate is, the more remarkable the effect of emission reduction will be. When the rate of carbon tax reaches 200 CNY/ton, the change rate of the total carbon emissions is −8.171%. Additionally, the higher the rate of carbon tax is, the more the tax revenue will be. As a particular tax with behavioral purpose, the carbon tax does not aim at increasing tax revenue. It aims at promoting the low-carbon transformation by passing on a marginal cost concept of emission reduction to society [73,74,75]. The carbon tax increased the cost of carbon emissions greatly. On one hand, it pushes high-carbon enterprises to carry out the innovation of low-carbon technology and improvement of technology. On the other hand, it guides enterprises to shift from passive emission reduction to active emission reduction by using more clean energy and less fossil energy, and improving the efficiency of fossil energy. 

## 5. Conclusions

Based on the “China’s non-competitive input–output table for 2018 (42 Departments)” and the carbon emission data of sectors provided by China Carbon Emission Accounts and Datasets (CEADs), the “China’s non-competitive input–output table for 2018 for Carbon Emissions after Carbon Tax (28 Departments)” was compiled in this paper. Additionally, the effect of carbon tax on product price, economy, employment, and emission reduction was studied through the input–output model, with the variation of carbon tax rate from 10 to 200 CNY/ton. The conclusions arrived are as follows:

(1) Levying the carbon tax will raise the product prices of 28 sectors to varying degrees. The continuous increases in the carbon tax rate lead to an increase in the product prices of all of these sectors. The top five sectors whose product price rise the highest are, successively, Electric Power Station, Production and Supply, Heating, Smelting and Pressing of Metals, and Nonmetal Mineral Production.
(2) When the carbon tax is levied, the GDP, the number of employees, and the carbon emissions in 28 sectors will all decline to varying degrees. With the increase in carbon tax rate, the decline degree in GDP, the number of employees, and carbon emissions the top five sectors ranked from high to low are Electric Power, Heating, Production and Supply, Construction, and Nonmetal Mineral Products.
(3) The inflation rate has a positive correlation with the carbon tax rate, while the change rate of the GDP and the change rate of carbon emission have a negative correlation with the carbon tax rate.
(4) Levying a low tax rate and establishing a dynamic adjustment mechanism is recommended, considering the impact of carbon tax on inflation and GDP, as well as the pressure of the current economic downturn.
(5) The EU carbon border regulation mechanism (i.e., carbon tariff) will be implemented from 1 October 2023, which means that China’s carbon price needs to be consistent with the international level in the future. The introduction of carbon tax is an effective measure for China to deal with the European and American carbon border regulation mechanism (CBAM).

## Figures and Tables

**Table 1:** 2018 Non-competitive input–output tables of China for carbon emissions after carbon tax; (calculated at producer’s price) Unit: 10,000 CNY.

Input-Output			Intermediate Use		Final Use + Carbon Tax	Total Domestic Output + Carbon Tax Or Import
			Energy Sector	Non-Energy Sector		
			1, 2, 3, 4	5, 6, …, 28		
Interme-diate input of domestic products	Energy Sector	1. Coal Mining and Dressing CO_2_ Emissions (10,000 Tons)				
		2. Petroleum and Natural Gas Extraction CO_2_ Emissions (10,000 Tons)				
		3. Refined Petroleum and Nuclear Fuel Processing CO_2_ Emissions (10,000 Tons)				
		4. Production and Supply of Gas CO_2_ Emissions (10,000 Tons)				
	Non- Energy Sector	5. Farming, Forestry, Animal Husbandry, Fishery, and Water Conservancy				
		6				
		…				
		28				
Interme-diate Input of Imports	Energy Sector	1. Coal Mining and Dressing				
		2. Petroleum and Natural Gas Extraction				
		3. Refined Petroleum and Nuclear Fuel Processing				
		4. Production and Supply of Gas				
	Non- Energy Sector	5. Farming, Forestry, Animal Husbandry, Fishery, and Water Conservancy				
		6				
		…				
		28				
Added value	Remuneration of workers (V)					
	Net Production Tax (T)					
	Carbon Tax (T*)					
	Depreciation of Fixed Assets (D)					
	Operating Surplus (S)					
	The Added Value Totals after Carbon Tax (GDP′)					
Total Input + Carbon Tax						

References: Chen Qingneng [61] (2019) and the compilation method of “2018 Non-competitive Input–Output Tables of China”.

**Table 2:** The relationship between carbon tax and inflation rate.

Carbon Tax Rate (T*) (CNY/ton)	T* = 10	T* = 20	T* = 30	T* = 40	T* = 50	T* = 60	T* = 70	T* = 80	T* = 90	T* = 100
Inflation Rate (IR)	0.293%	0.586%	0.880%	1.174%	1.468%	1.762%	2.057%	2.352%	2.647%	2.942%
Carbon Tax Rate (T*) (CNY/ton)	T* = 110	T* = 120	T* = 130	T* = 140	T* = 150	T* = 160	T* = 170	T* = 180	T* = 190	T* = 200
Inflation Rate (IR)	3.238%	3.533%	3.829%	4.125%	4.422%	4.718%	5.015%	5.312%	5.610%	5.907%

**Table 3:** Relationship between carbon tax and GDP. Impact of different carbon tax rates on the changes of total GDP.

Carbon Tax Rate (T*) (CNY/ton)	T* = 10	T* = 20	T* = 30	T* = 40	T* = 50	T* = 60	T* = 70	T* = 80	T* = 90	T* = 100
Change Rate of Total GDP (ΔGDP*′)	−0.108%	−0.215%	−0.320%	−0.424%	−0.526%	−0.627%	−0.727%	−0.825%	−0.921%	−1.017%
Carbon Tax Rate (T*) (CNY/ton)	T* = 110	T* = 120	T* = 130	T* = 140	T* = 150	T* = 160	T* = 170	T* = 180	T* = 190	T* = 200
Change Rate of Total GDP (ΔGDP*′)	−1.111%	−1.204%	−1.296%	−1.387%	−1.477%	−1.566%	−1.653%	−1.740%	−1.826%	−1.910%

**Table 4:** Relationship between carbon tax and total labor force.

Carbon Tax Rate (T*) (CNY/ton)	T*10	T*20	T*30	T*40	T*50	T*60	T*70	T*80	T*90	T*100
Change Rate of Labor Force (ΔL*′)	−0.097%	−0.193%	−0.288%	−0.381%	−0.474%	−0.565%	−0.655%	−0.745%	−0.833%	−0.920%
Carbon Tax Rate (T*) (CNY/ton)	T*110	T*120	T*130	T*140	T*150	T*160	T*170	T*180	T*190	T*200
Change Rate of Labor Force (ΔL*′)	−1.006%	−1.092%	−1.176%	−1.260%	−1.343%	−1.425%	−1.506%	−1.586%	−1.665%	−1.744%

**Table 5:** Relationship between carbon tax and total carbon emissions. The impact of different carbon tax rates on the change of carbon dioxide emission.

Carbon Tax Rate (T*) (CNY/ton)	T* = 10	T* = 20	T* = 30	T* = 40	T* = 50	T* = 60	T* = 70	T* = 80	T* = 90	T* = 100
Change Rate of Total Carbon Dioxide Emission (ΔCO_2_*′)	−0.526%	−1.035%	−1.530%	−2.011%	−2.477%	−2.931%	−3.372%	−3.801%	−4.218%	−4.625%
Carbon Tax Rate (T*) (CNY/ton)	T* = 110	T* = 120	T* = 130	T* = 140	T* = 150	T* = 160	T* = 170	T* = 180	T* = 190	T* = 200
Change Rate of Total Carbon Dioxide Emission (ΔCO_2_*′)	−5.021%	−5.406%	−5.782%	−6.149%	−6.507%	−6.855%	−7.196%	−7.529%	−7.854%	−8.171%

## Data Availability

Data will be made available on request.
